# Nitrogen-Containing Heterocycles as Significant Molecular Scaffolds for Medicinal and Other Applications

**DOI:** 10.3390/molecules26154617

**Published:** 2021-07-30

**Authors:** Éva Frank, György Szőllősi

**Affiliations:** 1 Department of Organic Chemistry, University of Szeged, Dóm tér 8, 6720 Szeged, Hungary; 2 Stereochemistry Research Group, Eötvös Loránd Research Network, University of Szeged, Eötvös utca 6, 6720 Szeged, Hungary

Most of the organic compounds applied as pharmaceuticals or intermediates utilized in their synthesis contain heterocyclic motifs. Among these, *aza*-heterocyclic derivatives are privileged scaffolds in the composition of biologically active natural and synthetic molecules. Due to their outstanding importance and their large variety both as concerns their structure and application possibilities, research oriented to novel preparation methods, transformations, multifaceted characterizations and applications in various fields still did not ease. A large number of books, monographs, journals and reviews are dedicated to publish discoveries concerning both the chemistry and the applicability of nitrogen-heterocyclic compounds. Research results are published continuously in journals dealing with organic, macromolecular, bioorganic and medicinal chemistry. Accordingly, an issue compiling communications on the preparation of novel compounds and application directions of *N*-heterocyclic scaffold containing molecules could be published on a regular base in any organic chemistry journal. This motivated us to edit the present issue, of which topic was not narrowed to special classes of *N*-heterocyclic compounds or a specific application area, rather we encouraged the submission and publication of novel, ingenious developments both in the synthesis and utilization of these materials, without excluding reviews complied on recent results.

Research reports published in the present issue covered a large area of *N*-heterocyclic compounds of various structure designed for miscellaneous purposes. Moreover, we have received and accepted for publication a manuscript reporting the preparation of pentafluorosulfanyl substituted *meta*-diamides [1]. Although these compounds are not heterocyclic, due to the promising efficiency of these diamides as insecticides, demonstrating the applicability of the –SF_5_ group instead of the isosteric –CF(CF_3_)_2_ substituent, and the presence of the former group in various biologically active *N*-heterocyclic compounds, we thought them appropriate for the communication for this Special Issue.

We were happy to see that the studies received incorporated a large structural diversity of the *N*-heterocyclic compounds. These included pyrrole, thiazole, pyrazole, benzimidazole, pyridine, pyrimidine, triazine and acridine derivatives, for most of them with some biological activity being unraveled. Thus, several compounds were synthetized with a benzimidazole scaffold connected to five-membered heterocycles, among which the 2-pyrrole derivative was found the most efficient as concerns its antioxidant, antifungal and antiproliferative activity, especially against human melanoma cells [2]. New paracyclophane derivatives bearing thiazole moieties were prepared and their anticancer activities were evaluated using nine human cancer cell lines [3]. Complete cell death was obtained with one of the synthetized compounds. In the same work, docking studies were used to predict the possible binding modes and interactions between the prepared paracyclophane derivatives and β-tubulin at the binding site of the enzyme. Several arylpyrazole derivatives and pyrazolocoumarin hybrids of estradiol were synthetized starting from estron-3-methyl ether and results of their anticancer evaluation was presented on different cell lines [4]. The phenyl substituted pyrazole ring containing compounds were found to be potent and tumor cell-selective anticancer agents on cervical and prostate cancer cell lines. Six pyrazolo[3,4-*d*]pyrimidines were prepared and their antimicrobial properties against a Gram-positive and a Gram-negative bacterium were examined [5]. As these types of compounds are known for their anticancer activity, the present results may be a starting point towards their application in the treatment of bacterial infections in cancer diagnosed patients.

Several papers published in this issue presented the synthesis and application of six membered *N*-heterocyclic derivatives, such as the N-substituted diamino-pyrimidine derivatives destined to act as novel anoctamin 1 ion channel blockers [6]. The preparation of twenty 1,3,5-triazine hydrazone derivatives was reported using a simple procedure by reacting aromatic aldehydes with 6-hydrazino-2,4-disubstituted-s-triazines. Structure–activity relationships were investigated evidencing that substituents both on the triazine scaffold and on the benzylidene moiety affect the antiproliferative activity of these compounds [7]. Another study reported the synthesis of ethyl 2-amino-4,5,6,7-tetrahydrobenzo[*b*]thiophene-3-carboxylate derivatives and pyrimidines or pyrimidinones prepared from these compounds. Examination of their apoptosis-inducing effect for breast cancer both in vitro and in vivo showed that a compound lacking *N*-heterocyclic moiety most effectively induced apoptosis and necrosis in the examined cancer cells; however, some pyrimide ring bearing materials also gave promising results as concerns their antitumor activity evaluations [8]. Interesting diversities on the properties and applicabilities of *N*-heterocyclic compounds were reported in two communications [9,10]. In one report, tosylated tetraaza-macrocyclic pyridinophane was used to evidence various supramolecular forces, which contribute to the stabilization of anionic species (Br_3_^−^), by analyzing the X-ray diffractograms of single crystals that grew from an aqueous acidic solution [9]. In the other communication, lipophilic enantiopure acridino-crown ethers were synthetized and used as efficient enantioselective carrier and sensor molecules in phase transport and electrochemical processes [10].

To our delight, three reviews were also submitted for publication in this Special Issue. One collected the 1,3,4-thiadiazole derivatives with potential anticancer activities, which were reported in the last ten years [11], the other surveyed recent developments in the synthesis of β-carboline alkaloids and related heterocyclic derivatives and their pharmacological potentials through examples published in the last five years [12]. In addition, significant results from the last decade on the synthetic modifications of pentacyclic triterpenoids with fused nitrogen-containing heterocyclic scaffolds and the anticancer activities of these molecular hybrids were also summarized [13]. Finally, our hopes cherished when lunching this Special Issue were fulfilled by the diversity of the novel *N*-heterocyclic compounds with various applications appeared in the communications published. The success of this issue confirmed that valuable results could be published in this area on a regular basis and encouraged us to consider editing other Special Issues on this vast research area in the future.
